# Estimating Functional Independence Measure Scores From Japanese Rehabilitation Notes Using a Large Language Model: Retrospective Study

**DOI:** 10.2196/100624

**Published:** 2026-10-27

**Authors:** Tomohiro Nishiyama, Yudai Mine, Norihide Itoh, Tatsuyoshi Ikenoue, Keima Nagasaka, Naoko Matsuo, Onishi Tatsuki, Eiji Aramaki

**Affiliations:** 1 Division of Information Science Nara Institute of Science and Technology Ikoma, Nara Japan; 2 Department of Rehabilitation Medicine Saiseikai Moriyama Municipal Hospital Moriyama, Shiga Japan; 3 Faculty of Rehabilitation School of Health Sciences Fujita Health University Toyoake, Aichi Japan; 4 Department of Public Health Faculty of Medicine University of Miyazaki Miyazaki, Miyazaki Japan; 5 Faculty of Data Science Shiga University Hikone, Shiga Japan

**Keywords:** functional independence measure, large language model, electronic medical record, rehabilitation, natural language processing, medical language processing.

## Abstract

**Background:**

The Functional Independence Measure (FIM) is a standard functional assessment tool in rehabilitation medicine; however, formal assessments are often conducted only at limited intervals. Free-text clinical notes contain more frequent observations of patients’ functional status and may complement these assessments.

**Objective:**

This study aimed to estimate item-level FIM scores from Japanese free-text rehabilitation notes using a locally deployed large language model (LLM) and to evaluate estimation performance; item-level estimability; and differences across diseases, therapy types, and admission FIM severity groups.

**Methods:**

We retrospectively analyzed free-text notes from physical therapy (PT), occupational therapy (OT), and speech-language pathology (SLP) for patients hospitalized with cerebral infarction (CeI), hip fracture (HF), or vertebral fracture (VBF) at Saiseikai Moriyama Municipal Hospital between October 2019 and September 2024. Using zero-shot prompting, an LLM estimated all FIM items from each note. Estimated scores were matched to reference FIM scores recorded for the same patient on the same calendar date. Performance was evaluated using the mean absolute error (MAE), root mean squared error, and quadratic weighted kappa. Estimation rates and comparisons with modal-score and last-observation-carried-forward (LOCF) baselines were also examined.

**Results:**

Among 2,952,702 eligible note-item pairs from 164,039 retained rehabilitation-note-level outputs, the model produced 980,000 valid estimates, corresponding to an overall estimation rate of 33.2%. Among 170,744 note-item opportunities with an available same-day reference score, 57,246 (33.5%) were estimable, of which 77.7% (44,481/57,246) were within 1 point of the reference score. Estimates derived from OT notes had a lower MAE than those derived from PT notes in CeI and HF, whereas the difference was not significant in VBF. The LLM outperformed the modal-score baseline but had a higher MAE than the LOCF overall (0.825 vs 0.648).

**Conclusions:**

The locally deployed LLM shows the potential to derive item-level FIM estimates from Japanese rehabilitation notes. Among estimable note-item pairs with a same-day reference FIM score, 77.7% were within 1 point of the reference score. These findings support the potential use of LLM-based estimation to structure functional information contained in routine rehabilitation notes and complement periodic FIM assessments. Further integration of multidisciplinary clinical records and external validation are needed to improve estimability and evaluate its value for longitudinal functional monitoring.

## Introduction

The Functional Independence Measure (FIM) is an internationally used scale for quantitatively assessing activities of daily living (ADLs) and is regarded as one of the standard outcome measures in rehabilitation medicine [1,2]. FIM plays an important role in discharge planning, prognostic estimation, and evaluation of intervention effects and has been applied across a wide range of disease areas, including stroke and orthopedic conditions [3-7].

However, the frequency of FIM assessment in clinical practice is subject to institutional and practical constraints. In many health care systems, its implementation is influenced by reimbursement schemes and operational restrictions. In addition, because FIM includes multiple items and requires structured scoring, repeated assessment may impose practical burdens in routine care. As a result, FIM is not necessarily conducted at a high frequency in routine clinical practice. Indeed, large-scale real-world clinical databases have reported that FIM is often collected only at limited time points within a clinical episode and frequently contains missing values, indicating difficulty in performing high-frequency assessments in real-world settings [8]. In Japan, a study reported that the introduction of FIM as a routine clinical task itself is treated as an intervention, indicating that high-frequency FIM assessment has not yet been fully established as part of usual care [9]. Moreover, even after the 2024 revision of the medical fee schedule, the minimum required frequency of FIM assessment is once every 2 weeks, and FIM assessments in routine practice are often conducted at this minimum interval, which is insufficient for high-frequency tracking of functional recovery during hospitalization [10]. Thus, although FIM is an important outcome measure, it tends to be sparse when treated as longitudinal data, limiting its ability to capture patients’ day-to-day subtle changes and functional capacity trajectories.

In contrast, free-text clinical notes that are routinely documented in rehabilitation practice have been reported to accumulate detailed observational records regarding patients’ functional status and therapeutic activities throughout rehabilitation interventions [11]. These narratives constitute a rich information source that could potentially complement FIM; however, because they are recorded as natural language, their systematic analysis and use are not straightforward.

In recent years, numerous natural language processing (NLP) approaches have been proposed to extract ADL-related information from free-text clinical notes [12]. As an early example, Anzaldi et al [13] applied a rule-based NLP approach to identify specific expressions documented in free-text clinical notes and analyzed their associations with geriatric syndromes. Regarding studies that extract information on ADLs and functional status from free-text clinical notes, Newman-Griffis et al [14] formulated the identification of mobility-related descriptions in rehabilitation records as a named entity recognition (NER) task [14], whereas Chen et al [15] reported NER-based extraction methods targeting geriatric syndromes, such as gait difficulty and urinary incontinence.

More recently, large language models (LLMs) have been applied directly to the extraction of functional status from clinical notes. Liu et al [16] evaluated an open-source LLM for extracting mobility status and classifying functional impairment across multiple institutions, whereas Kaster et al [17] used multiple LLMs to classify verbal and ambulatory abilities from clinical notes. These studies demonstrate the feasibility of LLM-based functional status extraction from free-text clinical notes. However, both conventional NLP approaches and recent LLM-based studies have primarily focused on individual functional domains or broad classification tasks rather than the comprehensive estimation of a standardized assessment instrument comprising multiple items and ordinal scores. Thus, a substantial gap remains between extracting ADL-related concepts from clinical text and quantifying functional status according to the detailed evaluation criteria and granularity used in clinical assessment scales, such as FIM.

Separate from text-based information extraction, several studies have also reported methods for estimating or predicting FIM scores. In these studies, structured clinical information, such as admission FIM scores and patient age, or motion sensor data are used as inputs, and machine learning or deep learning models are primarily used to predict discharge FIM scores [18,19]. However, these approaches do not directly use free-text clinical notes as input and are generally based on supervised learning. Due to privacy concerns and operational constraints, the requirement for task-specific training poses a substantial barrier to implementation in real-world clinical settings [20]. In many health care settings, it is difficult to export patient data outside the institution, and sufficient computational resources for fine-tuning LLMs within hospitals are often unavailable [21]. Therefore, there is a growing need for methods that can directly process existing free-text clinical notes without task-specific model training data while relying only on limited computational resources [22].

In this study, we focused on rehabilitation notes as a type of free-text clinical notes. Specifically, we investigated whether an LLM can estimate longitudinal FIM scores directly from Japanese free-text rehabilitation notes in a zero-shot setting, without task-specific training. To this end, we used rehabilitation notes to estimate all 18 FIM items at multiple time points and evaluated estimation performance across three disease categories—cerebral infarction (CeI), hip fracture (HF), and vertebral fracture (VBF)—and three therapy types: physical therapy (PT), occupational therapy (OT), and speech-language pathology (SLP). In addition, by stratifying analyses according to admission FIM severity and the item-level estimation rate, we examined how therapy-specific differences in documentation content affect estimation performance. Figure 1 summarizes this study design and evaluation framework. Beyond demonstrating feasibility, the findings of this study will contribute by identifying therapy-specific performance differences and by characterizing the error structure associated with documentation bias and differences in observational context.

**Figure 1:**
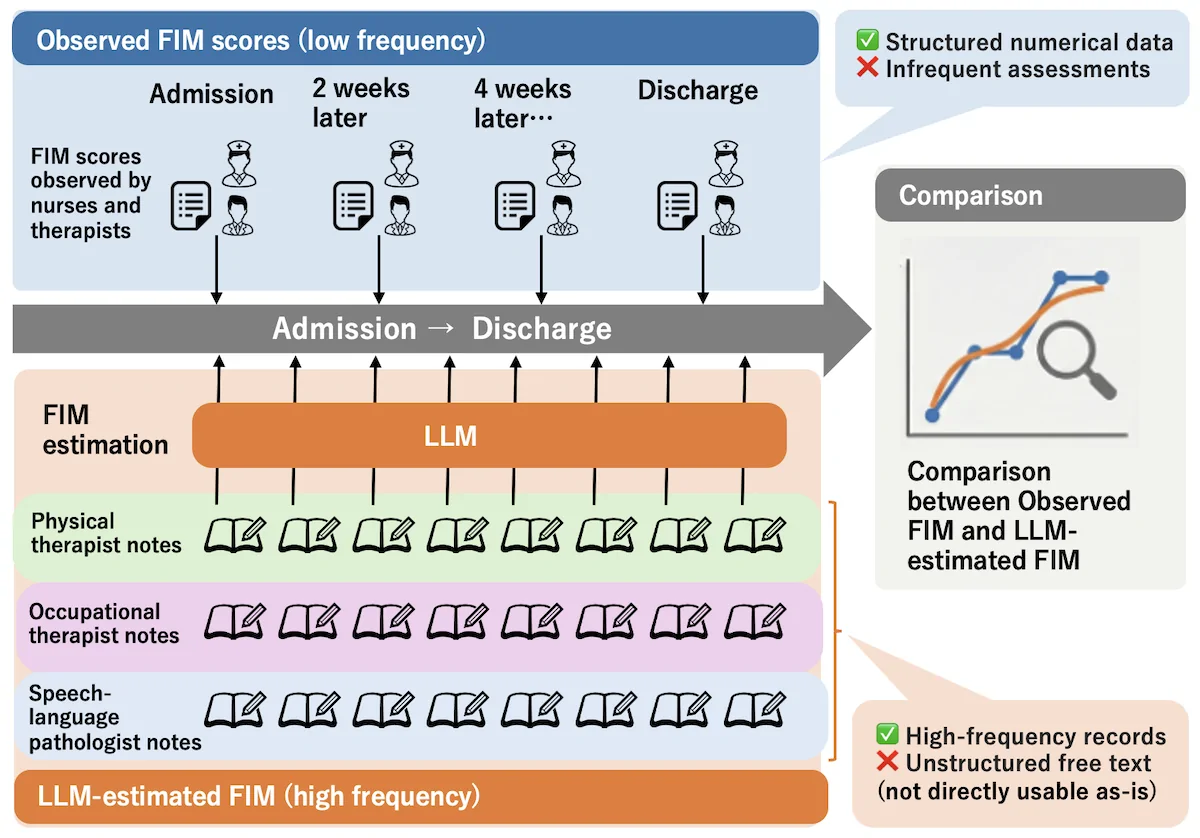
Study design and evaluation framework. An LLM was used to estimate patients’ FIM scores based on rehabilitation notes from PT, OT, and SLP. The estimated FIM scores were evaluated by comparison with the corresponding observed FIM scores. FIM: Functional Independence Measure; LLM: large language model; OT: occupational therapy; PT: physical therapy; SLP: speech-language pathology.

## Methods

### Data Collection

In this study, we analyzed patients aged 65 years or older who were admitted for rehabilitation purposes to Saiseikai Moriyama Municipal Hospital between October 2019 and September 2024, using Japanese rehabilitation notes from electronic health records (EHRs). The target conditions included three diseases: CeI, HF, and VBF. For each patient, rehabilitation notes were collected for up to 180 days after admission for CeI and up to 90 days after admission for HF and VBF. These observation periods were determined based on the disease-specific maximum lengths of stay eligible for reimbursement under Japan’s medical fee schedule for convalescent rehabilitation wards [23].

The extracted dataset included 233 patients with CeI, 461 patients with HF, and 148 patients with VBF, with 66,509, 74,056, and 23,474 rehabilitation notes, respectively. Observed FIM scores were measured at admission, approximately every 2 or 4 weeks during hospitalization, and at discharge. Because SLP is not routinely provided for fracture-related conditions in clinical practice, records related to this therapy type were excluded from the analysis for HF and VBF.

### LLM-Based Estimation of FIM Scores From Free-Text Rehabilitation Notes

Using an LLM, we estimated scores for all 18 FIM items at multiple time points from rehabilitation notes for patients with CeI, HF, and VBF. FIM consists of a total of 18 items, including 13 motor items and 5 cognitive items. The motor items include domains related to self-care (eating, grooming, bathing, upper body dressing, lower body dressing, and toileting), sphincter control (bladder management and bowel management), transfers (bed/chair/wheelchair transfers, toilet transfers, and tub/shower transfers), and locomotion (walking/wheelchair and stairs). The cognitive items include communication (comprehension and expression) and social cognition (social interaction, problem solving, and memory). Each FIM item is evaluated on a 7-point ordinal scale ranging from 1 (total assistance) to 7 (complete independence), with higher scores indicating greater functional independence [24].

We selected gemma-2-9b-it because its open weights and relatively compact model size allowed inference to be performed entirely within the hospital’s secured computing environment, while supporting Japanese-language input. The choice was primarily based on these operational requirements rather than on a dedicated benchmark of Japanese clinical-text tokenization or terminology.

Using this model, we estimated FIM scores from free-text rehabilitation notes through zero-shot prompting (Multimedia Appendix 1), as illustrated in Figure 2. The model was provided with each rehabilitation note and a condensed seven-level FIM scoring rubric and was instructed to output scores in a predefined JSON format. If the model determined that a specific FIM item could not be inferred from the note, the corresponding item was treated as unestimable. Each rehabilitation note was submitted to the model separately and was not concatenated with other notes recorded on the same day. For primary accuracy analyses, each estimable item score from each note was retained as a separate note-item observation and, when available, matched to the unique reference FIM item score recorded for the same patient on the same calendar date. If different reference scores were recorded for the same patient-date-item group, that group was excluded from the accuracy and agreement analyses.

**Figure 2:**
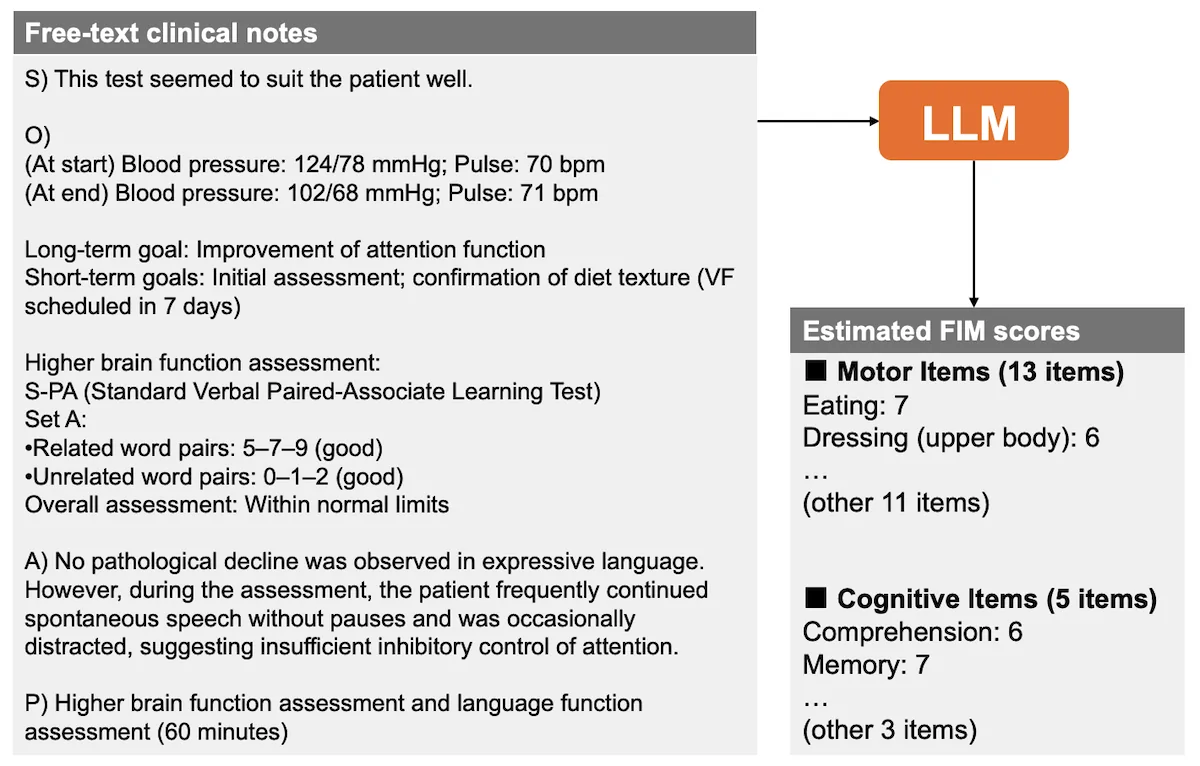
The LLM estimated FIM scores from Japanese rehabilitation notes, including 13 motor items and 5 cognitive items derived from patient-related narrative descriptions. FIM: Functional Independence Measure; LLM: large language model.

The study model was google/gemma-2-9b-it, loaded using the Hugging Face Transformers text-generation pipeline with bfloat16 weights and without 4- or 8-bit quantization. Inference used greedy decoding (do_sample=False) with a maximum of 256 generated tokens. Because sampling was disabled, temperature, top-p, and top-k were not applied, and no generation seed was used. No repetition penalty was explicitly specified; all other generation parameters used the model and library defaults. The maximum context length was 8192 tokens, including the input and generated output. The inference code did not explicitly truncate or split notes that exceeded the context limit. If an inference exception occurred, the affected note did not produce an output and was skipped.

### Evaluation

We evaluated estimation performance using the mean absolute error (MAE); mean error (ME), which preserves the sign of the difference; and root mean squared error (RMSE), which is more sensitive to large errors. Because FIM is an ordinal scale, we also computed quadratic weighted kappa (QWκ) as a measure of clinical agreement. The aggregate MAE, ME, and RMSE were microaveraged over all items and cases, and weighted kappa with quadratic weights was computed over all pooled item-level observations within each group.

Each estimated score was compared with the observed FIM score recorded for the same patient on the same date. Error metrics were calculated for item-level predictions with valid scores from 1 to 7. Outputs indicating nonestimable items were excluded from error metric calculations. The estimation rate was defined as the proportion of eligible note-item pairs for which the model produced a valid score and was reported for each item-by-therapy cell.

### Statistical Analysis

To account for the nonindependence of repeated observations within patients, we estimated 95% CIs for the prespecified performance metrics (MAE, ME, RMSE, and QWκ) using a patient-level cluster bootstrap with 2000 resamples, in which patients were resampled with replacement. Group comparisons between therapy types, motor and cognitive items, and disease groups were expressed as differences in the MAE and evaluated using the same patient-level cluster bootstrap approach. Two-sided *P* values for the 11 prespecified group comparisons were adjusted using the Benjamini-Hochberg false discovery rate procedure. Additional exploratory analyses were conducted. Model performance was stratified by sex and age group within each disease cohort, with the MAE and ME and 95% CIs estimated using the same patient-level cluster bootstrap procedure. Age groups were defined as <75, 75-84, and ≥85 years. For the last-observation-carried-forward (LOCF) comparison, LOCF-eligible pairs were additionally stratified by time since the previous FIM assessment.

To contextualize estimation accuracy, we compared the model with two naive baselines: (1) an empirical modal-score predictor based on the most frequent reference score for each disease-item combination and (2) an LOCF predictor using the most recent observed FIM score for the same patient and item before the index assessment date in the available longitudinal record, irrespective of admission episode. Prior FIM scores from previous admission episodes were retained to maximize the number of evaluable pairs and to apply the LOCF rule consistently using the most recent available FIM assessment. For each comparison, the LLM and the corresponding baseline were evaluated on the same eligible note-item pairs. Differences in MAEs between the LLM and each baseline, with 95% CIs, were estimated using the same patient-level cluster bootstrap approach. LOCF-eligible pairs were further stratified according to whether the current reference FIM score differed from the preceding observed score.

### Ethical Considerations

This study was approved by the Ethics Committee of Saiseikai Moriyama Municipal Hospital (approval #30; approval date November 20, 2024). As this was a retrospective observational study using secondary data from medical records, informed consent was handled through an opt-out approach via public disclosure on the hospital website. Patient identifiers used for data linkage were replaced with hashed identifiers and were not included in the model input. The approved study protocol included the use of clinical information from medical records, including chart records related to rehabilitation care. Before LLM inference, the free-text records were not subjected to automated de-identification or manual redaction. Inference was performed entirely within the hospital using a locally deployed model, and the notes were not transmitted to any external server or third-party model provider. Access to the unredacted notes was restricted to study authors who had authorized on-site access to the local computer at Saiseikai Moriyama Municipal Hospital, and these data were stored on a local computer within the hospital. Model outputs used for subsequent analysis consisted of numerical estimates without direct patient identifiers and were accessible to the study investigators. As an additional privacy-protection measure, after inference, the notes were manually screened for personal names using occupational titles and terms denoting family or social relationships, including abbreviations and English expressions, as search cues; identified names were removed. The accuracy and failure rate of this manual screening procedure were not formally evaluated. No compensation was provided to participants. This study did not include any images or other materials containing identifiable individuals. The study was conducted in accordance with the Declaration of Helsinki and relevant ethical guidelines.

## Results

### Overview

The results are presented in four parts. First, we describe the study cohort and the matched accuracy analysis set. Second, we report output validity and item-level estimation rates by disease, therapy type, and FIM item. Third, we present overall estimation accuracy and agreement, including therapy- and domain-specific comparisons. Finally, we examine estimation performance across admission FIM item-score groups.

### Study Cohort and Patient Characteristics

After applying disease-specific observation periods and adjudicating the rehabilitation target condition for the identified overlapping records, a total of 164,039 retained rehabilitation-note-level outputs were available for 842 patients: 66,509 (40.5%) rehabilitation notes from 233 (27.7%) patients with CeI, 74,056 (45.1%) notes from 461 (54.8%) patients with HF, and 23,474 (14.3%) notes from 148 (17.6%) patients with VBF (Table 1).

**Table 1:** Source rehabilitation data, patient characteristics in the matched accuracy analysis set, and same-day matched rehabilitation notes by disease type.

Characteristics		CeI^a^	HF^b^	VBF^c^
**Source rehabilitation data**				
	Patients with rehabilitation notes in the disease-specific observation period (N=842), n (%)	233 (27.7)	461 (54.8)	148 (17.6)
	Rehabilitation notes in the observation period (N=164,039), n (%)	66,509 (40.5)	74,056 (45.1)	23,474 (14.3)
	PT^d^ notes, n/N (%)	29,670/66,509 (44.6)	59,680/74,056 (80.6)	19,325/23,474 (82.3)
	OT^e^ notes, n/N (%)	21,248/66,509 (31.9)	14,376/74,056 (19.4)	4149/23,474 (17.7)
	SLP^f^ notes, n/N (%)	15,591/66,509 (23.4)	—^g^	—
**Matched accuracy analysis set**				
	Patients in the analysis set, n/N (%)	231/233 (99.1)	459/461 (99.6)	147/148 (99.3)
	Female sex, n/N (%)	104/231 (45.0)	371/459 (80.8)	106/147 (72.1)
	Male sex, n/N (%)	127/231 (55.0)	88/459 (19.2)	41/147 (27.9)
	Age at admission (years), mean (SD)	80.2 (7.0)	85.4 (7.6)	84.0 (6.4)
	Total FIM^h^ score at admission, mean (SD)	65.1 (29.0)	71.0 (24.1)	68.0 (23.6)
	Same-day matched rehabilitation notes^i^, n/N (%)	3559/66,509 (5.4)	4475/74,056 (6.0)	1463/23,474 (6.2)
	Admission-to-discharge FIM assessment interval^j^ (days), mean (SD)	78.2 (47.7)	56.8 (22.1)	50.3 (24.7)

^a^CeI: cerebral infarction.

^b^HF: hip fracture.

^c^VBF: vertebral fracture.

^d^PT: physical therapy.

^e^OT: occupational therapy.

^f^SLP: speech-language pathology.

^g^Not applicable.

^h^FIM: Functional Independence Measure. The total FIM score at admission was available for 222/231 (96.1%) patients with CeI, 447/459 (97.4%) patients with HF, and 141/147 (95.9%) patients with VBF.

^i^Rehabilitation notes were considered matched when a reference FIM assessment was available for the same patient on the same calendar date. Because multiple rehabilitation notes could be recorded on the same patient-date, these counts represent matched rehabilitation notes rather than unique FIM assessments.

^j^The admission-to-discharge FIM assessment interval was calculated as the number of days between admission and discharge FIM assessment dates and was calculable for 214/231 (92.6%) patients with CeI, 426/459 (92.8%) patients with HF, and 130/147 (88.4%) patients with VBF.

As shown in Table 1, the retained note-level outputs were subsequently matched to observed FIM assessments from the same patient on the same calendar date. Of the patients with retained outputs, 231 (99.1%) with CeI, 459 (99.6%) with HF, and 147 (99.3%) with VBF had at least one matched note and constituted the matched accuracy analysis set; the corresponding numbers of matched note records were 3559 (5.4%), 4475 (6.0%), and 1463 (6.2%), respectively.

**Output Validity and Estimation Rates**

Before matching the note-level outputs to observed FIM assessments, 164,039 retained rehabilitation-note-level outputs were available for the evaluation of output validity and item-level estimability. All 164,039 outputs were successfully parsed in JSON format. Separately, 42 execution-level exceptions produced no retained output and were excluded from the structured-output audit and subsequent matching. The median rehabilitation note length was 454 tokens (maximum 3056). No model input exceeded the 8192-token context limit, and none of the 42 execution-level exceptions were attributable to input length (Table S10 in Multimedia Appendix 2). There were 2,952,702 eligible note-item pairs (18 FIM items per note). The model produced 980,000 valid item-level estimates, corresponding to an overall estimation rate of 33.2%. The disease-specific estimation rates were 28.8% (344,597/1,197,162) for CeI, 35.9% (478,782/1,333,008) for HF, and 37.1% (156,621/422,532) for VBF. Detailed output validity results by disease are provided in Table S5 in Multimedia Appendix 2.

Table 2 presents the proportion of cases in which the LLM was able to estimate each FIM item, stratified by disease and therapy type. Overall, clear differences were observed in the distribution of estimable FIM items across therapy categories, whereas the overall distribution patterns were largely consistent across diseases. PT and OT showed high estimation rates for motor items related to transfers and locomotion, whereas SLP showed lower estimation rates for motor items and relatively higher estimation rates for cognitive items, such as comprehension and expression. These findings indicate that therapy-specific differences in documentation content were reflected in item-level estimability.

**Table 2:** LLM^a^ estimation rates of item-level FIM^b^ estimation by disease and therapy type^c^.

Category and FIM items		CeI^d^			HF^e^			VBF^f^	
		PT^g^ notes (n=29,670)	OT^h^ notes (n=21,248)	SLP^i^ notes (n=15,591)	PT notes (n=59,680)	OT notes (n=14,376)	PT notes (n=19,325)		OT notes (n=4149)
**Motor items, n (%)**									
	Eating	12,666 (42.7)	4738 (22.3)	3977 (25.5)	31,803 (53.3)	3997 (27.8)	10,660 (55.2)		1486 (35.8)
	Grooming	9589 (32.3)	5006 (23.6)	482 (3.1)	25,000 (41.9)	2877 (20.0)	8535 (44.2)		1055 (25.4)
	Bathing	9841 (33.2)	3139 (14.8)	503 (3.2)	25,793 (43.2)	2922 (20.3)	8605 (44.5)		1285 (31.0)
	Upper body dressing	11,120 (37.5)	8203 (38.6)	443 (2.8)	28,237 (47.3)	5016 (34.9)	9935 (51.4)		1938 (46.7)
	Lower body dressing	11,009 (37.1)	7772 (36.6)	319 (2.0)	27,753 (46.5)	4844 (33.7)	9661 (50.0)		1594 (38.4)
	Toileting	24,445 (82.4)	14,885 (70.1)	659 (4.2)	53,810 (90.2)	11,771 (81.9)	17,571 (90.9)		3345 (80.6)
	Bladder management	1096 (3.7)	2,027 (9.5)	105 (0.7)	2055 (3.4)	387 (2.7)	1012 (5.2)		199 (4.8)
	Bowel management	1174 (4.0)	1536 (7.2)	145 (0.9)	2199 (3.7)	199 (1.4)	1014 (5.2)		165 (4.0)
	Bed/chair/wheelchair transfer	28,243 (95.2)	17,958 (84.5)	1018 (6.5)	57,026 (95.6)	12,569 (87.4)	18,457 (95.5)		3571 (86.1)
	Toilet transfer	20,489 (69.1)	11,159 (52.5)	215 (1.4)	44,610 (74.7)	8242 (57.3)	14,866 (76.9)		2561 (61.7)
	Tub/shower transfer	131 (0.4)	2166 (10.2)	0	1282 (2.1)	1396 (9.7)	324 (1.7)		524 (12.6)
	Walking/wheelchair	28,065 (94.6)	15,015 (70.7)	1009 (6.5)	58,536 (98.1)	11,870 (82.6)	18,845 (97.5)		3311 (79.8)
	Stairs	4468 (15.1)	684 (3.2)	2 (0.0)	7956 (13.3)	241 (1.7)	2755 (14.3)		65 (1.6)
**Cognitive items, n (%)**									
	Comprehension	10,162 (34.3)	6916 (32.5)	12,286 (78.8)	17,841 (29.9)	2647 (18.4)	5128 (26.5)		787 (19.0)
	Expression	9408 (31.7)	6551 (30.8)	12,665 (81.2)	16,585 (27.8)	2410 (16.8)	4847 (25.1)		761 (18.3)
	Social interaction	116 (0.4)	3375 (15.9)	3002 (19.3)	472 (0.8)	1386 (9.6)	105 (0.5)		386 (9.3)
	Problem solving	103 (0.3)	1482 (7.0)	4690 (30.1)	376 (0.6)	674 (4.7)	65 (0.3)		242 (5.8)
	Memory	397 (1.3)	2080 (9.8)	5863 (37.6)	2498 (4.2)	1502 (10.4)	606 (3.1)		355 (8.6)
Mean (%)^j^		34.2	30.0	16.9	37.6	29.0	38.2		31.6

^a^LLM: large language model.

^b^FIM: Functional Independence Measure.

^c^Each cell shows the number of rehabilitation notes for which the LLM produced an estimate for the corresponding FIM item, with the percentage of analyzed notes for that therapy type shown in parentheses. Column denominators are shown in the header.

^d^CeI: cerebral infarction.

^e^HF: hip fracture.

^f^VBF: vertebral fracture.

^g^PT: physical therapy.

^h^OT: occupational therapy.

^i^SLP: speech-language pathology.

^j^The arithmetic mean of the percentages across the 18 FIM items.

During the reference data audit, 52 otherwise eligible note-item pairs arising from 21 patient-date-item groups involving 6 patients were excluded because more than one distinct reference FIM score was recorded for the same patient, calendar date, and item.

For the matched accuracy evaluation, 170,744 note-item opportunities had an available same-day reference FIM score. The LLM produced an estimate for 57,246 (33.5%) of these opportunities, whereas 113,498 (66.5%) were nonestimable. Accuracy and agreement metrics were calculated conditionally on the estimable note-item pairs.

### Overall Performance

As shown in Table 3, estimates derived from OT notes generally had lower MAE and RMSE values and higher QWκ values than those derived from PT notes across the three disease cohorts. In the CeI cohort, SLP-based estimates showed the largest estimation errors. The ME was positive overall and in the PT and OT strata, indicating mild overestimation on average, although the direction of error varied by admission FIM item-score group.

**Table 3:** FIM^a^ score estimation performance stratified by disease and therapy type^b^.

Disease and therapies			MAE^c^ (95% CI)		ME^d^ (95% CI)		RMSE^e^ (95% CI)		QWκ^f^ (95% CI)
**CeI^g^**									
	PT^h^	0.902 (0.832 to 0.973)		0.300 (0.221 to 0.375)		1.409 (1.336 to 1.481)		0.762 (0.725 to 0.796)	
	OT^i^	0.794 (0.728 to 0.859)		0.231 (0.152 to 0.308)		1.305 (1.232 to 1.377)		0.783 (0.750 to 0.812)	
	SLP^j^	1.159 (1.047 to 1.270)		–0.011 (–0.173 to 0.156)		1.632 (1.520 to 1.746)		0.636 (0.563 to 0.700)	
**HF^k^**									
	PT	0.909 (0.864 to 0.957)		0.183 (0.124 to 0.241)		1.430 (1.384 to 1.478)		0.664 (0.638 to 0.689)	
	OT	0.825 (0.762 to 0.888)		0.102 (0.029 to 0.179)		1.361 (1.292 to 1.429)		0.693 (0.652 to 0.732)	
**VBF^l^**									
	PT	0.923 (0.849 to 0.995)		0.171 (0.070 to 0.271)		1.428 (1.351 to 1.505)		0.682 (0.629 to 0.727)	
	OT	0.818 (0.723 to 0.913)		0.116 (–0.012 to 0.241)		1.298 (1.183 to 1.405)		0.711 (0.639 to 0.768)	

^a^FIM: Functional Independence Measure.

^b^Values are shown as point estimates with 95% CIs estimated using patient-level cluster bootstrap.

^c^MAE: mean absolute error.

^d^ME: mean error.

^e^RMSE: root mean squared error.

^f^QWκ: quadratic weighted kappa.

^g^CeI: cerebral infarction.

^h^PT: physical therapy.

^i^OT: occupational therapy.

^j^SLP: speech-language pathology.

^k^HF: hip fracture.

^l^VBF: vertebral fracture.

Patient-level cluster bootstrap comparisons with Benjamini-Hochberg correction showed significantly lower MAEs for OT- than for PT-based estimates in the CeI and HF cohorts but not in the VBF cohort (Table S2 in Multimedia Appendix 2). In the CeI cohort, both PT- and OT-based estimates had significantly lower MAEs than SLP-based estimates. Cognitive items had significantly higher MAEs than motor items in the CeI and HF cohorts, whereas no significant difference was observed in the VBF cohort. Overall MAEs did not differ significantly across the three disease cohorts.

Among the 57,246 estimable note-item pairs, 45.9% (26,285/57,246; 95% CI 44.5%-47.3%) exactly matched the reference FIM score, 31.8% (18,196/57,246) differed by exactly 1 point, and 22.3% (12,765/57,246) differed by 2 or more points. Accordingly, 77.7% (44,481/57,246; 95% CI 76.6%-78.8%) pairs were within 1 point of the reference score. When all 170,744 matched note-item opportunities with an available reference score were used as the denominator, including nonestimable items as unsuccessful estimation opportunities, the exact-match and within-one-point yields were 15.4% (26,285/170,744) and 26.1% (44,481/170,744), respectively. Item-specific agreement and confusion structures are presented in Table S4 in Multimedia Appendix 2 and Figure S1 in Multimedia Appendix 3.

Domain-specific performance metrics, including QWκ with patient-level cluster bootstrap 95% CIs, are reported in Table S8 in Multimedia Appendix 2. QWκ was numerically lower for cognitive than for motor items in the CeI (0.660 vs 0.777) and VBF (0.592 vs 0.692) cohorts, whereas the values were similar between domains in the HF cohort (0.682 vs 0.669).

Performance stratified by sex and age group within each disease cohort is presented in Table S9 in Multimedia Appendix 2. MAEs were similar between female and male patients across all three disease cohorts. Across age groups, MAEs tended to increase with age in the CeI and HF cohorts, whereas no clear age-related pattern was observed in the VBF cohort.

### Estimation Performance Stratified by Admission FIM Scores

Table 4 presents estimation performance stratified by admission FIM item-score group. Across all diseases, higher admission FIM scores were associated with smaller estimation errors, as reflected by lower MAE and RMSE values. This pattern was consistently observed in all three cohorts. Consistent with these findings, the paired marginal distributions showed that LLM-estimated scores tended to be higher than reference scores in the lower-admission-score group and slightly lower in the higher-admission-score group, although the magnitude and pattern varied across FIM items and diseases (Figure S2 in Multimedia Appendix 3).

**Table 4:** FIM^a^ estimation performance stratified by admission FIM item-score group.

Disease and admission FIM item-score groups		MAE^b^ (95% CI)		ME^c^ (95% CI)		RMSE^d^ (95% CI)		QWκ^e^ (95% CI)
**CeI^f^**								
	Total or maximal assistance	1.096 (1.018 to 1.178)	0.708 (0.611 to 0.808)		1.643 (1.563 to 1.727)		0.614 (0.566 to 0.654)	
	Moderate or minimal assistance	0.912 (0.845 to 0.976)	0.136 (0.038 to 0.242)		1.327 (1.252 to 1.396)		0.583 (0.523 to 0.635)	
	Supervision or higher	0.738 (0.660 to 0.818)	–0.140 (–0.218 to –0.068)		1.224 (1.137 to 1.313)		0.580 (0.510 to 0.630)	
**HF^g^**								
	Total or maximal assistance	1.308 (1.239 to 1.370)	0.758 (0.672 to 0.845)		1.884 (1.818 to 1.944)		0.546 (0.514 to 0.581)	
	Moderate or minimal assistance	0.878 (0.830 to 0.932)	0.146 (0.064 to 0.231)		1.293 (1.234 to 1.355)		0.556 (0.506 to 0.602)	
	Supervision or higher	0.661 (0.609 to 0.715)	–0.164 (–0.229 to –0.102)		1.119 (1.054 to 1.185)		0.478 (0.438 to 0.516)	
**VBF^h^**								
	Total or maximal assistance	1.179 (1.083 to 1.282)	0.725 (0.599 to 0.865)		1.741 (1.633 to 1.851)		0.650 (0.582 to 0.705)	
	Moderate or minimal assistance	0.967 (0.885 to 1.062)	0.110 (–0.090 to 0.304)		1.348 (1.246 to 1.463)		0.561 (0.478 to 0.633)	
	Supervision or higher	0.700 (0.616 to 0.791)	–0.198 (–0.322 to –0.092)		1.152 (1.045 to 1.268)		0.497 (0.435 to 0.547)	

^a^FIM: Functional Independence Measure.

^b^MAE: mean absolute error.

^c^ME: mean error.

^d^RMSE: root mean squared error.

^e^QWκ: quadratic weighted kappa.

^f^CeI: cerebral infarction.

^g^HF: hip fracture.

^h^VBF: vertebral fracture.

### Comparison With Naive Baselines

As shown in Table 5, the LLM was compared with two naive baselines using the same set of samples within each comparison. For the per item most-frequent-score baseline comparison (57,246/170,744, 33.5%), the LLM achieved substantially a lower MAE in every disease cohort (overall MAE 0.901 vs 1.661). For the LOCF baseline comparison (n=43,718), LOCF had a lower error (overall MAE 0.648 vs 0.825). Among the 43,718 LOCF-eligible note-item pairs, 28,472 (65.1%) had an unchanged reference FIM score from the previous assessment, whereas 15,246 (34.9%) had a changed reference FIM score. The LLM showed similar MAEs in the unchanged and changed subsets (0.821 and 0.834, respectively). In the changed subset, the LLM had a lower MAE than LOCF (MAE 0.834 vs 1.859). Most changed pairs represented improvement (13,833/15,246, 90.7%).

**Table 5:** LLM^a^ performance compared with baselines (MAE^b^; patient-level cluster bootstrap).

Comparison	Subset	Note-item pairs, n	MAE (LLM)	MAE (baseline)	ΔMAE (95% CI)
LLM vs most-frequent-score baseline	All retained matched estimable pairs	57,246	0.901	1.661	−0.761 (−0.812 to −0.706)
LLM vs LOCF^c^	All LOCF-eligible pairs	43,718	0.825	0.648	0.177 (0.133 to 0.219)
LLM vs LOCF	Observed FIM^d^ changed	15,246	0.834	1.859	−1.025 (−1.079 to −0.973)
LLM vs LOCF	Observed FIM stable	28,472	0.821	0.000	0.821 (0.779 to 0.859)

^a^LLM: large language model.

^b^MAE: mean absolute error.

^c^LOCF: last-observation-carried-forward.

^d^FIM: Functional Independence Measure.

### Comparison by FIM Item

To further examine differences in estimation performance across therapy categories, item-level estimation errors were analyzed by disease and therapy type. Tables S6 and S7 in Multimedia Appendix 2 present the MAE and ME for each FIM item. These item-level error patterns should be interpreted with caution for items with limited sample sizes, particularly in some therapy- and disease-specific subgroups (Table S1 in Multimedia Appendix 2). Overall, relatively smaller errors were observed for several mobility- and transfer-related items, whereas larger errors were observed for bathing-related, sphincter control, and some cognitive items, depending on therapy type.

## Discussion

### Principal Findings

#### Summary

This study demonstrated both the potential and the limitations of LLM-based FIM score estimation from rehabilitation notes. The model generated estimates for 33.2% of eligible note-item pairs, and 77.7% of estimable pairs were within 1 point of the same-day reference score. Estimability varied substantially by FIM item and therapy type, indicating that the information available for scoring depends on the documentation content. OT-derived estimates generally showed numerically lower errors than PT-derived estimates, although the adjusted difference in MAEs was significant for CeI and HF but not for VBF. In the CeI cohort, SLP-derived estimates showed higher errors, whereas their estimability was concentrated on selected cognitive items. Finally, errors were larger for lower admission FIM item-score groups, with a tendency toward overestimation at lower scores and underestimation at higher scores. These findings indicate that estimation performance is conditional on both the source documentation and the patient’s level of functional independence.

#### Item-Level Interpretation

##### Physical Therapy

In evaluations based on PT, relatively small estimation errors were observed for several transfer-related items, particularly bed/chair/wheelchair and toilet transfers. These activities represent core evaluation targets in PT, and information regarding task performance and the required level of assistance is often explicitly documented in rehabilitation notes. However, performance varied across other mobility-related items, including walking or wheelchair use, stairs, and tub or shower transfers.

In addition, cognitive items, such as comprehension, expression, social interaction, problem solving, and memory, are typically documented by physical therapists only in relation to physical safety or the patient’s ability to follow exercise instructions. Because these descriptions are qualitative and context dependent rather than structured assessments of cognitive function, they may not provide sufficient information for the model to accurately estimate standardized scores, which may have contributed to the larger estimation errors observed for these items.

##### Occupational Therapy

In evaluations based on OT, relatively stable estimation errors were observed across diseases for items primarily related to personal ADLs, such as dressing, grooming, and toileting. These activities constitute the core of assessment and training in OT, and the frequent inclusion of concrete, ADL-oriented descriptions in rehabilitation notes may have contributed to the stability of estimation performance.

In contrast, bladder and bowel management showed a different pattern from other personal ADL items. Although toileting was frequently documented in OT notes, bladder and bowel management had substantially lower estimation rates (Table 2). This finding indicated that sphincter control information is not routinely captured in therapist-authored rehabilitation notes. These items are inherently system dependent, primarily governed by ward-level nursing protocols and medical interventions rather than therapist-led functional training. OT notes seldom contain the specific information needed to estimate these items, such as details on incontinence frequency or the level of assistance required for bladder and bowel management. In addition, item-level errors for bladder and bowel management should be interpreted cautiously because they were based on small samples and varied across disease subgroups. These findings indicate that sparse documentation of sphincter control information primarily limits the estimability of these items from OT notes.

Notably, estimation errors for mobility- and transfer-related items were comparable between OT and PT notes, or smaller for OT notes in some analyses, although these items are conventionally regarded as PT-focused domains. PT notes may emphasize mobility performance under structured rehabilitation conditions, whereas OT notes often describe mobility and transfers within specific ADL tasks, including the assistance required and relevant contextual constraints. Because FIM scores reflect independence in daily activities, such contextualized OT documentation may align more closely with the reference assessments.

##### Speech-Language Pathology

In evaluations based on SLP, estimation errors were generally larger overall. Focusing on cognitive items, comparable levels of estimation errors to those observed in OT and PT were found for items such as comprehension, expression, social interaction, problem solving, and memory, indicating the difficulty of consistently estimating these cognitive functions from rehabilitation notes.

One possible explanation is that SLP notes often center on specific training tasks, such as auditory comprehension exercises or oral reading practice, and their levels of achievement, with detailed descriptions of performance in task-specific contexts. In contrast, information regarding the extent to which these abilities are independently manifested in daily living situations, or how they influence communication with others, is not always explicitly documented. As a result, inferring FIM cognitive items, which assess functional independence in everyday life, from task-level performance descriptions is challenging, and this may have contributed to the observed estimation errors.

#### Comparison With LOCF

Overall, the LLM had a higher MAE than LOCF. However, the LLM showed similar MAEs in the stable and changed subsets, with no substantial difference in estimation error according to whether the FIM score had changed since the previous assessment. This may reflect a fundamental difference in the information used by the two approaches: LOCF directly relies on the previous FIM score, whereas the LLM estimates the current FIM score from functional information documented in rehabilitation notes at the same time point.

In the analysis stratified by elapsed time since the previous FIM assessment, LOCF had a lower MAE than the LLM in all three strata. The difference was statistically significant in the 1-14- and ≥29-day strata but not in the 15-28-day stratum (Table S11 in Multimedia Appendix 2). These findings indicate that LOCF remained a strong baseline across the elapsed-time strata. At the same time, the potential value of the approach may lie not only in replacing LOCF on the basis of predictive accuracy but also in its ability to derive standardized functional information from routinely documented free-text rehabilitation notes.

#### Overestimation and Underestimation Across FIM Score Levels

Systematic differences in estimation error were observed according to the level of functional independence. The LLM tended to overestimate FIM scores in the severe assistance group, whereas underestimation was observed in the higher-score groups. This trend was also reflected in the marginal distributions of the reference and LLM-estimated FIM scores across admission item-score groups, although its magnitude and pattern varied across diseases and FIM items (Figure S2 in Multimedia Appendix 3). Two possible contributing factors are differences in the clinical contexts in which ADLs were observed and statistical characteristics of prediction on the bounded 1-7 FIM scale.

One possible clinical explanation is a difference in the criteria and contexts used for evaluating ADLs. In clinical assessment, a distinction is sometimes made between performed ADLs (what patients actually do in daily life) and capable ADLs (what patients are able to do under optimal conditions). In addition, when assessing performed ADLs, if a patient’s ability fluctuates throughout the day, the more dependent level of performance is generally recorded [25]. In this study, the observed FIM scores used as the reference standard were primarily based on patients’ performance in ward settings, assessed by nurses and therapists, and were therefore intended to reflect performed ADLs. In contrast, rehabilitation notes reflected observations made primarily by therapists during rehabilitation sessions.

Accordingly, rehabilitation notes may have placed relatively greater emphasis on ADL performance observed under assisted, structured, or environmentally adjusted conditions than on patients’ usual performance in daily ward activities. This difference in observational context may have contributed to higher LLM-estimated scores, particularly among patients requiring substantial assistance.

A second possible explanation is that imperfect predictions tend to be less extreme than the observed scores on the bounded 1-7 FIM scale. Consequently, low observed scores are more likely to be overestimated, whereas high observed scores are more likely to be underestimated. The floor and ceiling constraints of the scale may further contribute to this pattern by limiting the possible direction of errors at the extremes. Therefore, both differences in observational context and the tendency of the LLM to produce less extreme predictions may have contributed to the observed pattern of estimation error.

A broader consideration in interpreting these estimation errors is that the reference FIM scores were based on routine clinical assessments jointly performed by nurses and therapists and may therefore include some degree of variability arising from differences in observation, professional perspective, and clinical judgment. Previous studies have reported high interrater reliability for the total FIM score, with a median reliability coefficient of 0.95. At the item level, reliability varied substantially, with generally higher reliability for motor items than for cognitive and communication items [2]. In this study, agreement between the LLM-estimated and reference item scores, assessed using pooled QWκ, was 0.713 (95% CI 0.694-0.731). This value cannot be directly compared with published estimates of human interrater reliability, because the measures differ in their statistical definitions, units of analysis, study populations, and assessment settings. Nevertheless, given the known variability in human FIM assessment, the observed disagreement likely reflects not only model prediction error but also some variability inherent in the reference assessment.

#### Need for Multisource Clinical Data Integration

FIM items related to sphincter control consistently showed low estimability by the LLM across all therapy categories and disease groups. Although sphincter control constitutes an essential component of FIM, information relevant to these items is often not systematically documented in the free-text notes written by physical therapists, occupational therapists, or speech-language pathologists.

This finding indicates that when only therapist-authored notes are used as input, the information required to estimate items that strongly depend on ward-level daily living and nursing care, such as sphincter control, is inherently missing from the text.

Therefore, to reconstruct FIM trajectories in a more comprehensive and accurate manner, it is necessary to integrate therapist-authored notes with nursing records and other sources of EHR data. For example, combining structured records of toileting patterns with observational descriptions documented in nursing records may help complement aspects of daily functioning that are difficult to capture using therapist notes alone. Overall, these results highlight the limitations of relying on single-profession clinical notes for LLM-based FIM score estimation and underscore the importance of multisource data integration strategies.

Previous clinical NLP research has shown that different types of clinical documents contain complementary patient information and yield different extraction patterns [26]. Consistent with these observations, PT, OT, and SLP notes should not be treated as interchangeable, and future multisource models should represent note source explicitly and evaluate source-specific performance.

In addition to integrating multiple data sources, future studies should consider the architecture used to convert clinical text into FIM scores. Although our zero-shot end-to-end approach reduces the need for task-specific annotation, intermediate representations, and rule development, providing a condensed FIM rubric does not ensure that each estimate is supported by the appropriate item-specific criteria or explicit evidence in the note. Previous clinical NLP studies have demonstrated both extraction-first approaches that map relevant clinical attributes to standardized clinical scores [27] and modular LLM pipelines that decompose clinical feature extraction into multiple stages [28]. Applied to FIM estimation, a similar two-stage pipeline could first extract relevant functional attributes and supporting text and then apply item-specific scoring rules. Such an approach could improve auditability and help distinguish extraction errors from scoring errors. However, it would require validated schemas and scoring rules and could propagate errors between stages. Direct comparison of end-to-end and structured-extraction-first approaches is therefore warranted. In addition to predictive accuracy, future evaluations should assess whether each estimate is supported by explicit evidence in the note and is consistent with the applicable item-specific FIM criteria.

### Limitations

This study has several limitations. First, it was conducted at a single institution and focused on specific disease groups in a convalescent rehabilitation ward; therefore, the generalizability of the findings should be interpreted with caution.

Second, the evaluation was based on a lightweight LLM intended for in-hospital deployment and a condensed 7-level FIM scoring rubric, rather than the full item-specific decision rules used in comprehensive FIM manuals. This may have limited fine-grained item-level scoring. In a small, partially overlapping supplementary pilot, the OT<PT<SLP ordering of the MAE was reproduced across the Gemma and Qwen prompt-model configurations, indicating that the therapy-specific pattern may not be unique to Gemma. However, Llama-3-ELYZA-JP-8B produced no usable estimates for the requested FIM item keys, and the limited pilot sample does not establish generalizability across lightweight LLMs. A supplementary comparison of gemma-2-9b-it with other comparably sized open models is presented in Multimedia Appendix 4.

Third, the analysis was limited to free-text notes written by therapists, and the performance of models integrating multidisciplinary records, such as nursing or physician notes, was not evaluated.

Fourth, accuracy analyses were restricted to rehabilitation notes recorded on the same day as an observed FIM assessment. Because documentation on assessment days may be more comprehensive than routine notes, the reported accuracy may overestimate performance in routine clinical documentation. In addition, because nonestimable item scores were not imputed, the model did not provide complete longitudinal trajectories. Although LOCF was evaluated as a naive comparator, carry-forward approaches may lag behind changes in functional status. Alternative approaches for handling missing estimates, including multiple or model-based imputation, remain to be evaluated in future studies.

Fifth, the study period overlapped with the COVID-19 pandemic, during which changes in infection control protocols, staffing, rehabilitation delivery, and documentation practices may have affected the data available for analysis. Because no notes or LLM estimates were generated when therapy was not provided, pandemic-related changes may have altered the patients and clinical situations represented in the analyzed data and introduced unmeasured temporal variation in estimation performance.

Finally, the clinically recorded FIM scores were used as the reference standard but should not be regarded as error-free ground truth. FIM scoring involves clinical judgment and inherent interrater variability, even when scores are determined through multidisciplinary assessment. Consequently, part of the disagreement attributed to the LLM may reflect variability in the reference FIM scores. Because independent duplicate ratings were unavailable, interrater reliability could not be estimated within this cohort. In addition, because blinded human ratings based solely on the same rehabilitation notes were unavailable, we could not distinguish model-specific errors from disagreement arising from differences in the observational contexts and functional information represented by therapist-authored notes and ward-based FIM assessments.

### Conclusion

The locally deployed LLM in this study showed the potential to derive item-level FIM estimates from Japanese rehabilitation notes. Among estimable note-item pairs with a same-day reference FIM score, 77.7% were within 1 point of the reference score. Performance and estimability varied across therapy types, FIM items, diseases, and admission functional severity, underscoring the importance of the type and content of the source documentation. These findings support the potential use of LLM-based estimation to structure functional information contained in routine rehabilitation notes and complement periodic FIM assessments. Further integration of multidisciplinary clinical records and external validation are needed to improve estimability and establish its utility for longitudinal functional monitoring and clinical workflow support.

## Data Availability

The data sets generated and analyzed during this study are not publicly available due to privacy and ethical restrictions.

## Appendix

Multimedia Appendix 1 Prompt used for FIM estimation. FIM: Functional Independence Measure.

Multimedia Appendix 2 Sample sizes for item-level FIM estimation analyses stratified by disease and therapy type; differences in MAE across therapy types, FIM domains, and disease cohorts; unique patients contributing evaluable note-item pairs to item-level error analyses, stratified by FIM item, disease, and therapy type; item-level agreement and error distribution of LLM-estimated FIM scores; item-level classification of retained LLM outputs before matching to reference FIM assessments, by disease; item-level MAE and ME of FIM score estimation stratified by disease and therapy type; FIM score estimation performance stratified by disease, FIM domain, sex, and age group within each disease cohort; distribution of input lengths in tokens and relation to the model context limit; and model and LOCF performance stratified by time since the previous FIM assessment. FIM: Functional Independence Measure; LLM: large language model; LOCF: last-observation-carried-forward; MAE: mean absolute error; ME: mean error.

Multimedia Appendix 3 Confusion matrices and marginal score distributions.

Multimedia Appendix 4 Prompt and model sensitivity analysis.

## Abbreviations

ADL: activity of daily living
CeI: cerebral infarction
EHR: electronic health record
FIM: Functional Independence Measure
HF: hip fracture
LLM: large language model
LOCF: last-observation-carried-forward
MAE: mean absolute error
ME: mean error
NER: named entity recognition
NLP: natural language processing
OT: occupational therapy
PT: physical therapy
QWκ: quadratic weighted kappa
RMSE: root mean squared error
SLP: speech-language pathology
VBF: vertebral fracture
