# Supplementary material for: Estimating Functional Independence Measure Scores From Japanese Rehabilitation Notes Using a Large Language Model: Retrospective Study
Source: JMIR Med Inform. 2026 Oct 27;14:e100624. doi: 10.2196/100624 (PMC13752589; doi:10.2196/100624)
Supplement: Supplementary file 1 [file medinform_v14i1e100624_app1.docx]

**Multimedia Appendix 1. Prompt Used for FIM Estimation**

The following textbox presents the prompt used for FIM estimation. The original Japanese prompt was used in the experiments.

Textbox S1. Prompt used for FIM estimation (original Japanese version).

リハビリテーションの医師が診断したカルテを与えます。

FIM(Functional Independence Measure)のスコアを予想してください。

\# 評価項目

【FIMの評価項目一覧】

▶︎ 運動項目（13項目）

セルフケア

(1)食事

(2)整容

(3)清拭

(4)更衣上半身

(5)更衣下半身

(6)トイレ動作

排泄コントロール

(7)排尿管理

(8)排便管理

移乗

(9)ベッド・椅子・車椅子移乗

(10)トイレ移乗

(11)浴槽・シャワー移乗

移動

(12)歩行・車椅子

(13)階段

▶︎ 認知項目（5項目）

コミュニケーション

(14)理解

(15)表出

社会的認知

(16)社会的交流

(17)問題解決

(18)記憶

\# 採点基準

▶︎ 運動項目の採点基準

◎7点(完全自立)

補助具または介助なしで「自立」して行える。

◎6点(修正自立)

時間が掛かる。装具や自助具、服薬が必要。安全性の配慮が必要。

◎5点(監視・準備)

監視、準備、指示、促しが必要。

◎4点(最小介助)

手で触れる以上の介助は必要ない。「75％以上」は自分で行う。

◎3点(中等度介助)

手で触れる以上の介助が必要。「50％〜75％未満」は自分で行う。

◎2点(最大介助)

「25％〜50％未満」は自分で行う。

◎1点(全介助)

「25％未満」しか自分で行わない。

▶︎ 認知項目の採点基準

◎7点(完全自立)

複雑な事項を「自立」して一人でできる。

◎6点(修正自立)

時間がかかる。投薬している。安全性の配慮が必要。

◎5点(監視・補助)

監視、準備、指示、促しが必要。簡単な事項に介助が10%未満必要。

◎4点(最小介助)

「75％以上90%未満」は自分で行う。

◎3点(中等度介助)

「50％〜75％未満」は自分で行う。

◎2点(最大介助)

「25％〜50％未満」は自分で行う

◎1点(全介助)

「25％未満」しか自分で行わない。

\# カルテ

{medical_record}

\# 出力

- 出力はjson形式でお願いします。

- 18の評価項目について、採点基準に従って1~7の数字で採点してください。

- カルテから評価が読み取れない項目については採点結果を-1としてください。

- 採点についてのコメントは不要です。jsonのみ出力してください。

出力フォーマット例を以下に示します。

{

"食事": ,

"整容": ,

"清拭": ,

"更衣上半身": ,

"更衣下半身": ,

"トイレ動作": ,

"排尿管理": ,

"排便管理": ,

"ベッド・椅子・車椅子移乗": ,

"トイレ移乗": ,

"浴槽・シャワー移乗": ,

"歩行・車椅子": ,

"階段": ,

"理解": ,

"表出": ,

"社会的交流": ,

"問題解決": ,

"記憶":

}

Textbox S2. Text added at the beginning of the original prompt for the persona-framed variant.

あなたは回復期リハビリテーション病棟で豊富な経験を持つリハビリテーション専門医です。以下の指示に従ってください。

Textbox S3. Exact text added at the end of the original prompt for the strengthened-output-constraints variant.

\# 重要な注意

- 必ず有効なJSONのみを出力してください（前後の説明文は一切不要）。

- 読み取れない項目は必ず -1 としてください。

- 18項目すべてを必ず含めてください。
