# Supplementary material for: Estimating Functional Independence Measure Scores From Japanese Rehabilitation Notes Using a Large Language Model: Retrospective Study
Source: JMIR Med Inform. 2026 Oct 27;14:e100624. doi: 10.2196/100624 (PMC13752589; doi:10.2196/100624)
Supplement: Supplementary file 2 [file medinform_v14i1e100624_app2.docx]

**Multimedia Appendix 2. Supplementary Tables**

Table S1. Sample sizes for item-level FIM estimation analyses stratified by disease and therapy type. Each cell denotes the number of matched samples used to compute the error metrics. A matched sample was defined as an item estimate paired with an observed FIM for the same patient on the same date.

|  | Disease | CI | CI | CI | HF | HF | VBF | VBF |
| --- | --- | --- | --- | --- | --- | --- | --- | --- |
|  | Therapy | PT | OT | SLP | PT | OT | PT | OT |
|  | FIM item |  |  |  |  |  |  |  |
| Motor items | Eating | 721 | 245 | 245 | 1970 | 273 | 715 | 122 |
|  | Grooming | 548 | 250 | 30 | 1523 | 191 | 562 | 89 |
|  | Bathing | 558 | 140 | 34 | 1559 | 186 | 567 | 99 |
|  | Upper body dressing | 616 | 389 | 29 | 1670 | 311 | 637 | 137 |
|  | Lower body dressing | 611 | 346 | 21 | 1656 | 294 | 622 | 118 |
|  | Toileting | 1284 | 748 | 49 | 3170 | 740 | 1075 | 225 |
|  | Bladder management | 80 | 81 | 9 | 146 | 28 | 72 | 12 |
|  | Bowel management | 88 | 45 | 10 | 154 | 17 | 70 | 13 |
|  | Bed/chair/wheelchair transfer | 1478 | 914 | 54 | 3412 | 783 | 1121 | 243 |
|  | Toilet transfer | 1120 | 565 | 20 | 2665 | 488 | 896 | 181 |
|  | Tub/shower transfer | 16 | 86 | – | 82 | 74 | 15 | 36 |
|  | Walking/wheelchair | 1474 | 814 | 57 | 3503 | 746 | 1142 | 229 |
|  | Stairs | 243 | 32 | – | 523 | 15 | 178 | 6 |
| Cognitive items | Comprehension | 514 | 333 | 696 | 1055 | 191 | 321 | 66 |
|  | Expression | 463 | 313 | 721 | 970 | 176 | 306 | 63 |
|  | Social interaction | 4 | 146 | 152 | 32 | 87 | 8 | 26 |
|  | Problem solving | 1 | 50 | 240 | 27 | 47 | 5 | 19 |
|  | Memory | 21 | 82 | 322 | 184 | 116 | 38 | 40 |

Table S2. Differences in mean absolute error across therapy types, FIM domains, and disease cohorts. ΔMAE was calculated as the MAE of the first group minus that of the second group; therefore, positive values indicate a higher MAE in the first group. The 95% confidence intervals and two-sided P values were estimated using a patient-level cluster bootstrap with 2,000 resamples. The P values were adjusted across all 11 comparisons using the Benjamini–Hochberg false discovery rate procedure.

| Cohort | Comparison | ΔMAE [95% CI] | q (BH-FDR) | Sig. |
| --- | --- | --- | --- | --- |
| HF | PT − OT | +0.084 [+0.017, +0.151] | 0.0385 | Yes |
| HF | motor − cognitive | −0.123 [−0.235, −0.017] | 0.0385 | Yes |
| CI | PT − OT | +0.108 [+0.022, +0.192] | 0.0385 | Yes |
| CI | PT − SLP | −0.257 [−0.385, −0.132] | 0.0018 | Yes |
| CI | OT − SLP | −0.365 [−0.476, −0.250] | 0.0018 | Yes |
| CI | motor − cognitive | −0.247 [−0.344, −0.153] | 0.0018 | Yes |
| VBF | PT − OT | +0.105 [−0.005, +0.205] | 0.1053 | No |
| VBF | motor − cognitive | −0.057 [−0.245, +0.139] | 0.7026 | No |
| HF vs CI | overall | −0.012 [−0.078, +0.052] | 0.8922 | No |
| HF vs VBF | overall | −0.009 [−0.089, +0.066] | 0.8976 | No |
| CI vs VBF | overall | +0.003 [−0.083, +0.088] | 0.9520 | No |

Table S3: Number of unique patients contributing evaluable note–item pairs to the item-level error analyses, stratified by FIM item, disease, and therapy type. Each cell shows the number of unique patients with at least one LLM-estimated item score paired with a reference FIM score from the same patient and calendar date. A patient was counted only once within each FIM item and disease–therapy stratum, regardless of the number of contributing notes or assessment dates. A dash indicates that no evaluable note–item pair was available.

| FIM item | CI-PT | CI-OT | CI-SLP | HF-PT | HF-OT | VBF-PT | VBF-OT |
| --- | --- | --- | --- | --- | --- | --- | --- |
| Eating | 178 | 108 | 97 | 389 | 131 | 129 | 41 |
| Grooming | 163 | 107 | 14 | 361 | 109 | 117 | 36 |
| Bathing | 159 | 80 | 20 | 360 | 109 | 118 | 40 |
| Upper body dressing | 172 | 140 | 20 | 385 | 152 | 127 | 53 |
| Lower body dressing | 170 | 127 | 15 | 384 | 154 | 125 | 49 |
| Toileting | 221 | 190 | 27 | 454 | 239 | 144 | 63 |
| Bladder management | 31 | 30 | 7 | 61 | 19 | 26 | 10 |
| Bowel management | 36 | 21 | 8 | 66 | 14 | 27 | 10 |
| Bed/chair/wheelchair transfer | 227 | 205 | 28 | 458 | 244 | 146 | 65 |
| Toilet transfer | 214 | 172 | 9 | 432 | 193 | 135 | 58 |
| Tub/shower transfer | 13 | 45 | – | 50 | 52 | 7 | 17 |
| Walking/wheelchair | 229 | 204 | 28 | 459 | 242 | 146 | 65 |
| Stairs | 99 | 18 | – | 189 | 12 | 54 | 5 |
| Comprehension | 129 | 100 | 194 | 251 | 103 | 72 | 29 |
| Expression | 120 | 95 | 196 | 241 | 95 | 71 | 27 |
| Social interaction | 3 | 48 | 51 | 20 | 45 | 4 | 12 |
| Problem solving | 1 | 19 | 97 | 17 | 26 | 3 | 7 |
| Memory | 14 | 37 | 134 | 85 | 71 | 23 | 20 |

Table S4: Item-level agreement and error distribution of LLM-estimated FIM scores.

| FIM item | n | Exact agreement, n (%) | Within ±1 point, n (%) | ≥2-point error, n (%) |
| --- | --- | --- | --- | --- |
| Eating | 4291 | 1797 (41.9) | 3156 (73.5) | 1135 (26.5) |
| Grooming | 3193 | 1411 (44.2) | 2389 (74.8) | 804 (25.2) |
| Bathing | 3143 | 1018 (32.4) | 2004 (63.8) | 1139 (36.2) |
| Upper body dressing | 3789 | 1674 (44.2) | 2961 (78.1) | 828 (21.9) |
| Lower body dressing | 3668 | 1549 (42.2) | 2867 (78.2) | 801 (21.8) |
| Toileting | 7291 | 3488 (47.8) | 5988 (82.1) | 1303 (17.9) |
| Bladder management | 428 | 316 (73.8) | 374 (87.4) | 54 (12.6) |
| Bowel management | 397 | 197 (49.6) | 360 (90.7) | 37 (9.3) |
| Bed/chair/wheelchair transfer | 8005 | 4075 (50.9) | 7114 (88.9) | 891 (11.1) |
| Toilet transfer | 5935 | 3067 (51.7) | 5201 (87.6) | 734 (12.4) |
| Tub/shower transfer | 309 | 113 (36.6) | 193 (62.5) | 116 (37.5) |
| Walking/wheelchair | 7965 | 3963 (49.8) | 5618 (70.5) | 2347 (29.5) |
| Stairs | 997 | 491 (49.2) | 774 (77.6) | 223 (22.4) |
| Comprehension | 3176 | 1182 (37.2) | 2172 (68.4) | 1004 (31.6) |
| Expression | 3012 | 1218 (40.4) | 2144 (71.2) | 868 (28.8) |
| Social interaction | 455 | 215 (47.3) | 334 (73.4) | 121 (26.6) |
| Problem solving | 389 | 187 (48.1) | 277 (71.2) | 112 (28.8) |
| Memory | 803 | 324 (40.3) | 555 (69.1) | 248 (30.9) |

Table S5. Item-level classification of retained LLM outputs before matching to reference FIM assessments, by disease. Note–item slots were calculated as the number of retained note-level outputs multiplied by 18. Scores from 1 to 7 were treated as valid estimates. The predefined non-estimable code (−1) denoted an item that could not be estimated from the note, whereas other out-of-range numeric values were treated as invalid. Neither category contributed to the numerator of the estimation rate, and both were excluded from the accuracy and agreement analyses.

| Disease | Retained outputs, N | Note–item slots, N | Scores 1–7, n/N (%) | The predefined non-estimable code (−1), n/N (%) | Other out-of-range values, n/N (%) |
| --- | --- | --- | --- | --- | --- |
| Cerebral infarction | 66,509 | 1,197,162 | 344,597/1,197,162 (28.8%) | 852,545/1,197,162 (71.2%) | 20/1,197,162 (0.002%) |
| Hip fracture | 74,056 | 1,333,008 | 478,782/1,333,008 (35.9%) | 854,203/1,333,008 (64.1%) | 23/1,333,008 (0.002%) |
| Vertebral fracture | 23,474 | 422,532 | 156,621/422,532 (37.1%) | 265,900/422,532 (62.9%) | 11/422,532 (0.003%) |
| Overall | 164,039 | 2,952,702 | 980,000/2,952,702 (33.2%) | 1,972,648/2,952,702 (66.8%) | 54/2,952,702 (0.002%) |

Table S6. Item-level MAE of FIM score estimation stratified by disease and therapy type. Values are not reported for cells with fewer than 10 matched note–FIM pairs.

|  | Disease | CI | CI | CI | HF | HF | VBF | VBF |
| --- | --- | --- | --- | --- | --- | --- | --- | --- |
|  | Therapy | PT | OT | SLP | PT | OT | PT | OT |
| Category | FIM item |  |  |  |  |  |  |  |
| Motor items | Eating | 1.019 | 0.914 | 1.629 | 1.016 | 0.996 | 1.106 | 1.016 |
|  | Grooming | 0.916 | 0.896 | 1.333 | 0.904 | 0.906 | 0.925 | 0.629 |
|  | Bathing | 1.154 | 1.107 | 1.412 | 1.243 | 1.376 | 1.473 | 1.232 |
|  | Upper body dressing | 0.834 | 0.763 | 1.103 | 0.875 | 0.781 | 0.984 | 0.818 |
|  | Lower body dressing | 0.843 | 0.671 | 1.333 | 0.928 | 0.840 | 0.994 | 0.661 |
|  | Toileting | 0.776 | 0.705 | 1.327 | 0.787 | 0.736 | 0.786 | 0.689 |
|  | Bladder management | 0.263 | 0.469 | – | 0.582 | 1.464 | 0.347 | 0.250 |
|  | Bowel management | 0.852 | 0.378 | 1.200 | 0.539 | 1.118 | 0.571 | 1.538 |
|  | Bed/chair/wheelchair transfer | 0.632 | 0.629 | 1.352 | 0.633 | 0.561 | 0.664 | 0.700 |
|  | Toilet transfer | 0.690 | 0.655 | 0.700 | 0.627 | 0.578 | 0.742 | 0.707 |
|  | Tub/shower transfer | 2.875 | 0.977 | – | 1.866 | 1.554 | 2.400 | 1.000 |
|  | Walking/wheelchair | 1.098 | 0.955 | 1.544 | 1.210 | 1.051 | 0.954 | 0.948 |
|  | Stairs | 1.062 | 0.375 | – | 0.927 | 0.867 | 1.129 | – |
| Cognitive items | Comprehension | 1.286 | 0.940 | 1.109 | 1.145 | 0.780 | 1.019 | 0.712 |
|  | Expression | 1.199 | 0.853 | 1.183 | 0.935 | 0.739 | 0.869 | 0.762 |
|  | Social interaction | – | 1.308 | 0.507 | 1.000 | 1.069 | – | 1.038 |
|  | Problem solving | – | 1.140 | 1.008 | 1.370 | 0.851 | – | 0.842 |
|  | Memory | 1.095 | 0.878 | 1.146 | 1.179 | 0.767 | 1.605 | 1.175 |

Table S7. Item-level ME of FIM score estimation stratified by disease and therapy type. Values are not reported for cells with fewer than 10 matched note–FIM pairs.

|  | Disease | CI | CI | CI | HF | HF | VBF | VBF |
| --- | --- | --- | --- | --- | --- | --- | --- | --- |
|  | Therapy | PT | OT | SLP | PT | OT | PT | OT |
|  | FIM item |  |  |  |  |  |  |  |
| Motor items | Eating | -0.626 | -0.212 | -0.649 | -0.768 | -0.740 | -0.972 | -0.754 |
|  | Grooming | -0.318 | 0.336 | 0.333 | -0.412 | -0.361 | -0.441 | -0.337 |
|  | Bathing | 0.871 | 0.764 | 1.000 | 0.786 | 0.763 | 1.155 | 0.848 |
|  | Upper body dressing | 0.023 | 0.198 | -0.552 | -0.227 | -0.158 | 0.071 | -0.088 |
|  | Lower body dressing | 0.277 | 0.185 | -0.381 | 0.077 | 0.071 | 0.244 | 0.119 |
|  | Toileting | 0.210 | 0.269 | -0.510 | 0.029 | 0.061 | 0.059 | 0.209 |
|  | Bladder management | 0.237 | 0.321 | – | 0.377 | 0.679 | 0.125 | 0.250 |
|  | Bowel management | 0.716 | 0.022 | -1.000 | 0.292 | -0.412 | 0.400 | -1.077 |
|  | Bed/chair/wheelchair transfer | 0.196 | 0.152 | 0.056 | 0.130 | 0.070 | 0.102 | 0.173 |
|  | Toilet transfer | 0.187 | 0.230 | -0.200 | 0.135 | 0.025 | 0.195 | 0.122 |
|  | Tub/shower transfer | 2.500 | 0.930 | – | 1.549 | 1.014 | 2.267 | 0.722 |
|  | Walking/wheelchair | 0.732 | 0.507 | 0.421 | 0.804 | 0.547 | 0.551 | 0.572 |
|  | Stairs | 0.379 | 0.000 | – | 0.270 | -0.067 | 0.388 | – |
| Cognitive items | Comprehension | 1.006 | -0.021 | 0.348 | 0.845 | -0.068 | 0.670 | -0.136 |
|  | Expression | 0.719 | -0.125 | -0.301 | 0.467 | 0.000 | 0.405 | -0.095 |
|  | Social interaction | – | 0.486 | -0.086 | 0.375 | 0.471 | – | 0.038 |
|  | Problem solving | – | 0.100 | 0.242 | 1.148 | -0.170 | – | -0.316 |
|  | Memory | 0.143 | -0.122 | 0.165 | 0.832 | 0.147 | 1.237 | 0.075 |

Table S8. FIM score estimation performance stratified by disease and FIM domain. Values are presented with 95% confidence intervals estimated using a patient-level cluster bootstrap with 2,000 resamples. Weighted kappa was calculated using quadratic weights.

| Disease | FIM domain | Note-item pairs, n | Patients, n | MAE [95% CI] | ME [95% CI] | RMSE [95% CI] | Quadratic weighted kappa [95% CI] |
| --- | --- | --- | --- | --- | --- | --- | --- |
| CI | Motor items | 14,050 | 231 | 0.852 [0.798, 0.908] | +0.229 [+0.171, +0.290] | 1.361 [1.303, 1.421] | 0.777 [0.747, 0.804] |
| CI | Cognitive items | 4,058 | 221 | 1.099 [1.003, 1.189] | +0.244 [+0.111, +0.376] | 1.583 [1.487, 1.669] | 0.660 [0.605, 0.712] |
| HF | Motor items | 26,179 | 459 | 0.883 [0.841, 0.924] | +0.129 [+0.072, +0.182] | 1.412 [1.368, 1.453] | 0.669 [0.645, 0.691] |
| HF | Cognitive items | 2,885 | 317 | 1.006 [0.896, 1.115] | +0.547 [+0.410, +0.685] | 1.482 [1.363, 1.597] | 0.682 [0.622, 0.734] |
| VBF | Motor items | 9,182 | 147 | 0.900 [0.834, 0.968] | +0.136 [+0.056, +0.216] | 1.404 [1.332, 1.474] | 0.692 [0.647, 0.731] |
| VBF | Cognitive items | 892 | 98 | 0.956 [0.771, 1.132] | +0.429 [+0.140, +0.684] | 1.431 [1.240, 1.610] | 0.592 [0.402, 0.733] |

Table S9. FIM score estimation performance stratified by sex and age group within each disease cohort.

| Disease | Subgroup | Patients, n | Note–item pairs, n | MAE [95% CI] | ME [95% CI] |
| --- | --- | --- | --- | --- | --- |
| CI | Female | 104 | 8,914 | 0.905 [0.816, 0.985] | +0.210 [+0.122, +0.302] |
| CI | Male | 127 | 9,194 | 0.910 [0.847, 0.974] | +0.254 [+0.173, +0.341] |
| CI | Age <75 years | 56 | 4,228 | 0.758 [0.652, 0.880] | +0.147 [+0.044, +0.265] |
| CI | Age 75–84 years | 116 | 9,268 | 0.928 [0.857, 1.000] | +0.200 [+0.103, +0.289] |
| CI | Age ≥85 years | 59 | 4,612 | 1.002 [0.910, 1.090] | +0.377 [+0.259, +0.498] |
| HF | Female | 371 | 23,781 | 0.894 [0.848, 0.943] | +0.153 [+0.092, +0.212] |
| HF | Male | 88 | 5,283 | 0.903 [0.819, 0.992] | +0.247 [+0.140, +0.349] |
| HF | Age <75 years | 54 | 3,342 | 0.672 [0.567, 0.791] | +0.129 [+0.011, +0.245] |
| HF | Age 75–84 years | 134 | 8,580 | 0.829 [0.753, 0.909] | +0.166 [+0.078, +0.246] |
| HF | Age ≥85 years | 271 | 17,142 | 0.972 [0.922, 1.023] | +0.180 [+0.107, +0.255] |
| VBF | Female | 106 | 7,445 | 0.904 [0.826, 0.989] | +0.135 [+0.033, +0.231] |
| VBF | Male | 41 | 2,629 | 0.905 [0.813, 1.003] | +0.239 [+0.087, +0.385] |
| VBF | Age <75 years | 14 | 1,078 | 0.904 [0.750, 1.052] | +0.204 [-0.112, +0.422] |
| VBF | Age 75–84 years | 59 | 3,753 | 0.851 [0.749, 0.958] | +0.125 [-0.017, +0.252] |
| VBF | Age ≥85 years | 74 | 5,243 | 0.943 [0.849, 1.040] | +0.179 [+0.059, +0.299] |

Table S10. Distribution of input lengths in tokens and relation to the model context limit.

| Input | Median [IQR], tokens | Maximum, tokens |
| --- | --- | --- |
| Rehabilitation note only | 454 [370–563] | 3056 |
| Full model input, including instructions and chat template | 1284 [1200–1393] | 3885 |

Table S11. Model and LOCF performance stratified by time since the previous FIM assessment.

| Time since previous FIM assessment | n | MAE (Model) | MAE (LOCF) | ΔMAE [95% CI] |
| --- | --- | --- | --- | --- |
| 1–14 days | 12,853 | 0.789 | 0.334 | +0.454 [+0.404, +0.505] |
| 15–28 days | 29,749 | 0.831 | 0.780 | +0.050 [−0.002, +0.105] |
| ≥29 days | 1,116 | 1.102 | 0.750 | +0.352 [+0.132, +0.569] |
