# Supplementary material for: Estimating Functional Independence Measure Scores From Japanese Rehabilitation Notes Using a Large Language Model: Retrospective Study
Source: JMIR Med Inform. 2026 Oct 27;14:e100624. doi: 10.2196/100624 (PMC13752589; doi:10.2196/100624)
Supplement: Supplementary file 3 [file medinform_v14i1e100624_app3.docx]

**Multimedia Appendix 3. Confusion Matrices and Marginal Score Distributions**

Figure S1. Item-level confusion matrices of LLM-estimated versus reference FIM scores. Rows indicate reference FIM scores, and columns indicate LLM-estimated scores. Cell labels show absolute counts, colors represent row-normalized percentages, and row denominators are shown to the right of each matrix. Rows based on fewer than 10 evaluable pairs were suppressed because their percentage distributions were considered unstable.

Figure S2. Marginal distributions of reference and LLM-estimated FIM scores by disease, FIM item, and admission item-score group. Within each stratum, the Reference and LLM blocks show the percentages of estimable note-item pairs assigned to scores 1 through 7 using a shared color scale; n denotes the common denominator for the paired distributions. Gray cells indicate strata with fewer than 10 estimable note-item pairs or no available pairs.
