# Supplementary material for: Estimating Functional Independence Measure Scores From Japanese Rehabilitation Notes Using a Large Language Model: Retrospective Study
Source: JMIR Med Inform. 2026 Oct 27;14:e100624. doi: 10.2196/100624 (PMC13752589; doi:10.2196/100624)
Supplement: Supplementary file 4 [file medinform_v14i1e100624_app4.docx]

**Multimedia Appendix 4. Prompt and Model Sensitivity Analysis**

## Methods

We performed a supplementary prompt- and model-sensitivity analysis using a pilot dataset comprising 39 patients and 234 rehabilitation notes linked to a week-4 FIM assessment. This pilot analysis was conducted after completion of the main analysis; therefore, its results were not used to select the model or the primary prompt used in the main analysis. Of the 39 patients, 28 were also included in the main analysis set. The pilot included hip fracture, intracerebral hemorrhage, and cerebral infarction cases and was intended for exploratory comparison of inference configurations.

We evaluated gemma-2-9b-it, Qwen2.5-7B-Instruct, and Llama-3-ELYZA-JP-8B using three prompt formulations: the original study prompt, a persona-framed variant that identified the model as an experienced rehabilitation physician, and a strengthened-output variant that reiterated the requirements for valid JSON, all 18 item keys, and -1 for non-inferable items. All configurations used greedy decoding (do_sample=False), bfloat16 weights without 4-bit or 8-bit quantization, and a maximum of 512 generated tokens. No generation seed was used because sampling was disabled.

For each model–prompt configuration, we calculated MAE, the item-level estimation rate, and the proportion of outputs from which a JSON object could be parsed.

## Results

For gemma-2-9b-it, overall MAE was similar under the original, persona-framed, and strengthened-output prompts (0.955, 0.967, and 0.950, respectively), while estimation rates ranged from 31.7% (1,336/4,212) to 37.1% (1,564/4,212). Qwen2.5-7B-Instruct showed greater variation in MAE (0.985–1.136), with estimation rates ranging from 17.6% (740/4,212) to 22.9% (963/4,212). Under the original prompt, gemma-2-9b-it had a lower MAE and a higher estimation rate than Qwen2.5-7B-Instruct (MAE, 0.955 vs 1.065; estimation rate, 32.3% [1,361/4,212] vs 19.6% [827/4,212], respectively). Across all six model–prompt configurations evaluated for Gemma and Qwen, OT notes yielded lower MAE than PT and SLP notes.

A JSON object could be parsed from all 234 outputs for each Gemma and Qwen configuration. For Llama-3-ELYZA-JP-8B, a JSON object could be parsed from 59/234 (25.2%), 70/234 (29.9%), and 7/234 (3.0%) outputs under the original, persona-framed, and strengthened-output prompts, respectively; however, none yielded usable estimates for the requested FIM item keys (Table S1).

Table S1. Prompt- and model-sensitivity analysis in a pilot dataset of 39 patients and 234 rehabilitation notes. Of the 39 patients, 28 were also represented in the main analysis set. Estimation rate was calculated across 4212 note-item opportunities (234 notes × 18 FIM items). A parseable JSON object was defined as an output from which a JSON object could be decoded; this did not by itself require all 18 requested item keys to contain usable scores. MAE was calculated only for item estimates from 1 to 7 with an available FIM reference. A dash indicates that no usable item estimates were produced. OT indicates occupational therapy; PT, physical therapy; and SLP, speech-language pathology.

| Model | Prompt variant | Parseable JSON objects, n/N (%) | Estimable note-item pairs, n/N (%) | Overall MAE | OT MAE (evaluable n) | PT MAE (evaluable n) | SLP MAE (evaluable n) |
| --- | --- | --- | --- | --- | --- | --- | --- |
| gemma-2-9b-it | Original | 234/234 (100.0) | 1,361/4,212 (32.3) | 0.955 | 0.852 (n=391) | 0.952 (n=897) | 1.548 (n=73) |
|  | Persona-framed | 234/234 (100.0) | 1,564/4,212 (37.1) | 0.967 | 0.879 (n=448) | 0.972 (n=1,025) | 1.341 (n=91) |
|  | Strengthened output constraints | 234/234 (100.0) | 1,336/4,212 (31.7) | 0.950 | 0.803 (n=386) | 0.975 (n=872) | 1.397 (n=78) |
| Qwen2.5-7B-Instruct | Original | 234/234 (100.0) | 827/4,212 (19.6) | 1.065 | 0.822 (n=332) | 1.150 (n=434) | 1.787 (n=61) |
|  | Persona-framed | 234/234 (100.0) | 963/4,212 (22.9) | 1.136 | 0.873 (n=363) | 1.231 (n=542) | 1.897 (n=58) |
|  | Strengthened output constraints | 234/234 (100.0) | 740/4,212 (17.6) | 0.985 | 0.791 (n=301) | 1.043 (n=396) | 1.814 (n=43) |
| Llama-3-ELYZA-JP-8B | Original | 59/234 (25.2) | 0/4,212 (0.0) | — | — | — | — |
|  | Persona-framed | 70/234 (29.9) | 0/4,212 (0.0) | — | — | — | — |
|  | Strengthened output constraints | 7/234 (3.0) | 0/4,212 (0.0) | — | — | — | — |

## Interpretation

This pilot analysis suggests that Gemma’s MAE was relatively stable across the tested prompt variants and that the therapy-specific pattern of lower MAE for OT than for PT and SLP was also observed with Qwen2.5-7B-Instruct. However, because the pilot sample was small, partially overlapped with the main analysis set, and differed in note selection, FIM reference timing, and disease composition, it should not be considered an independent validation of the therapy-specific performance patterns observed in the main analysis. The absolute performance estimates should also be interpreted cautiously.
